# Case Report: Contralateral radiculopathy associated with facet tropism after transforaminal lumbar interbody fusion

**DOI:** 10.3389/fsurg.2026.1837069

**Published:** 2026-09-10

**Authors:** Xiaowen Du, Jiangwei Tan

**Affiliations:** Spinal Surgery, Yantai Affiliated Hospital of Binzhou Medical University, Yantai, China

**Keywords:** contralateral radiculopathy, degenerative spondylolisthesis, facet tropism, foraminotomy, transforaminal lumbar interbody fusion

## Abstract

Contralateral radiculopathy is an infrequently reported postoperative complication in patients with degenerative spondylolisthesis (DS) who receive transforaminal lumbar interbody fusion (TLIF). Herein, we report two clinical cases with this complication associated with preoperative facet tropism. Case 1 involved a patient with L4/5 DS presenting with radiating pain in the right lower extremity; new-onset left lower limb weakness and radicular pain occurred immediately after TLIF via the right side. A revision resection of the left L5 superior articular process (SAP) led to marked improvement of the patient's symptoms. Case 2 was diagnosed with L4/5 DS, and this patient developed right lower-extremity pain and weakness after left-sided primary TLIF. The contralateral radicular symptoms were significantly relieved via percutaneous endoscopic foraminotomy. This complication arises from mechanical compression of the exiting spinal nerve root by a sagittalized SAP (associated with facet tropism) after complete segmental reduction in the two cases. Surgical revision is indicated once this adverse event is identified, and percutaneous endoscopic foraminotomy may be a minimally invasive option in selected patients and in experienced hands.

## Background

Facet tropism was first defined as angular asymmetry between bilateral lumbar facet joints, in which one facet exhibits a predominantly sagittal orientation relative to its contralateral counterpart (1). Masharawi et al. (2) demonstrated that facet angular asymmetry represents a normal anatomical feature of thoracic vertebrae rather than lumbar vertebrae, indicating that lumbar facet tropism is correlated with spinal degenerative lesions. Fujiwara et al. (3) documented that sagittalized facet joint morphology develops secondary to osteoarthritic remodeling or as a combined consequence of osteoarthritis and facet joint synovial effusion. Multiple clinical investigations have confirmed significant correlations between lumbar facet tropism, intervertebral disc herniation, and degenerative spondylolisthesis (DS) (4, 5).

DS is characterized by anterior slippage of one vertebral body over the caudal vertebral segment driven by progressive intervertebral and facet joint degeneration, while the posterior neural arch remains intact. This pathological slippage leads to spinal canal stenosis and the hallmark clinical presentation of neurogenic intermittent claudication (6). Transforaminal lumbar interbody fusion (TLIF) serves as a mainstream surgical intervention for DS, which achieves segmental lumbar interbody arthrodesis through unilateral or bilateral posterolateral surgical corridors (7). In recent decades, minimally invasive TLIF (MI-TLIF) and full-endoscopic TLIF (Endo-TLIF) have gained wide acceptance in DS treatment, as these techniques minimize paraspinal soft tissue trauma (8, 9).

Herein, we report two cases of patients who developed contralateral radiculopathy after TLIF for DS. We further explore the pathogenic mechanisms underlying this complication and identify facet tropism as a possible predisposing risk factor in both cases. We also propose corresponding intraoperative preventive and postoperative therapeutic strategies.

## Case presentations

### Case 1

A 54-year-old female without other underlying comorbidities apart from osteopenia [bone mineral density (BMD) −2.2] was hospitalized for a 6-month history of low back pain and radiating pain in the right lower extremity that failed to respond to standard conservative management such as bed rest, oral non-steroidal anti-inflammatory drugs, and neurotrophic agents [visual analog scale (VAS) 6, Oswestry disability index (ODI) 60]. The pain radiated from the right buttock to the lateral aspect of the right thigh and calf. The clinical timeline for the patient is presented in Table 1.

**Table 1:** Clinical timeline for the patient (case 1).

Time point	Key events
Admission day (day 0)	Admitted with a 6-month history of low back pain and right lower-extremity radiating pain refractory to conservative treatment (VAS 6, ODI 60). A physical examination showed right foot dorsum hypoesthesia and right extensor hallucis longus strength grade 4, with a positive right straight leg raise test result
Hospital day 1 X-ray, CT, and MRI performed	Lumbar radiographs demonstrated L4/5 grade Ⅰ spondylolisthesis (Figure 1); An MRI confirmed L4/5 disc herniation and right foraminal stenosis caused by a sequestered disc fragment (Figure 2). A CT revealed L4/5 facet degeneration, elongated bilateral L5 superior articular processes (SAPs), and facet tropism (Figure 3)
Hospital day 2 (day of primary surgery)	Underwent open L4/5 TLIF via a right-sided approach with cement-augmented fenestrated pedicle screw fixation
Hospital day 3 (CT and MRI performed conservative management)	New-onset left anterolateral thigh radiating pain and left medial calf dysesthesia emerged (VAS 7). An examination revealed left quadriceps strength grade 4 and diminished left patellar tendon reflex. A postoperative CT showed a satisfactory implant position without cement leakage but an anterior displacement of the residual left L5 SAP leading to secondary left foraminal stenosis (Figure 4). An MRI excluded epidural hematoma and residual disc compression (Figure 5). The patient responded poorly to conservative treatment such as analgesics, non-steroidal anti-inflammatory drugs, and low-dose steroids
Hospital day 9 (day of revision surgery)	Underwent open revision surgery to resect the residual left L5 SAP and achieve left neuroforaminal decompression (Figure 6). Left radicular pain was markedly alleviated after the revision operation (VAS 3)
Hospital day 12	The patient was discharged in a satisfactory condition
Six-month follow-up	Left quadriceps muscle strength recovered to grade 5
One-year follow-up	Left medial calf numbness fully resolved (ODI 10). Dynamic radiographs confirmed solid interbody fusion. No recurrent radiculopathy or implant-related complications occurred, and the patient achieved favorable recovery

#### Physical examination and imaging studies

A physical examination revealed hypoesthesia over the right dorsum of the foot and grade 4 muscle strength of the right extensor hallucis longus, while the muscle strength of other bilateral limb muscles was normal. The straight leg raise test was positive on the right side. Bilateral patellar tendon reflexes and Achilles tendon reflexes were normal, and the bowel and bladder functions were intact.

**Figure 1:**
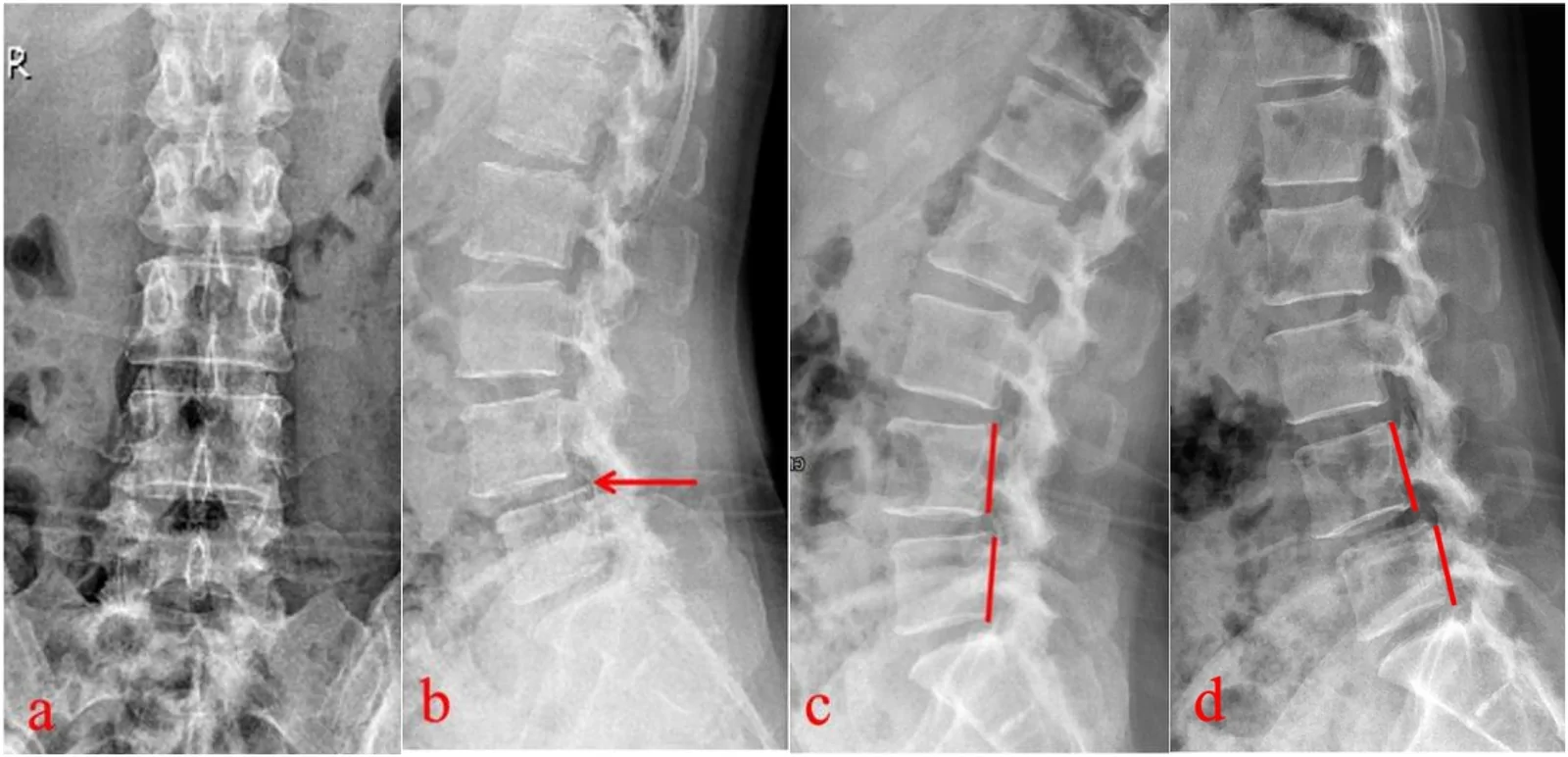
Preoperative lumbar radiographs demonstrated grade Ⅰ spondylolisthesis at the level of L4/5 on the lateral view **(b)** without scoliosis on the anteroposterior (AP) view **(a)** the lateral dynamic views revealed segmental instability at the L4/5 level **(c,d)**.

**Figure 2:**
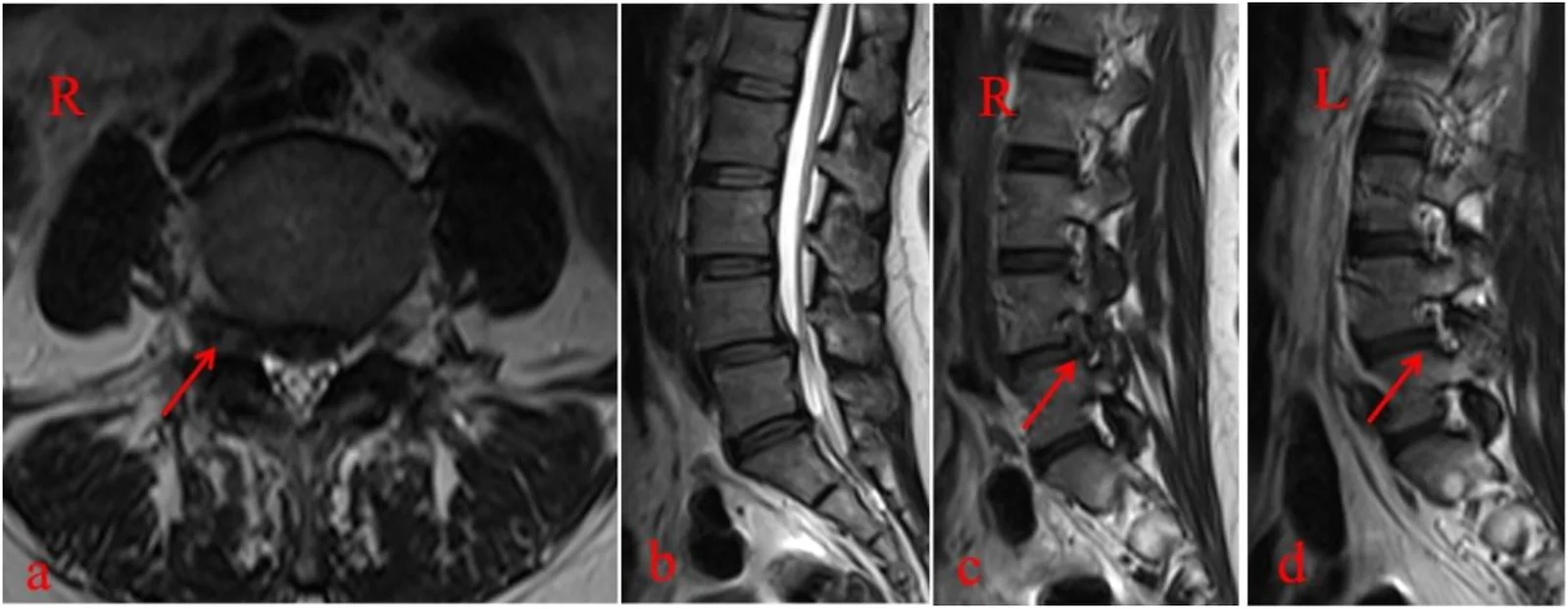
A preoperative MRI revealed L4/5 disc herniation **(a,b)** and right foraminal stenosis secondary to a sequestered disc fragment **(a,c)**, with the left foramen appearing nearly normal **(d).**

**Figure 3:**
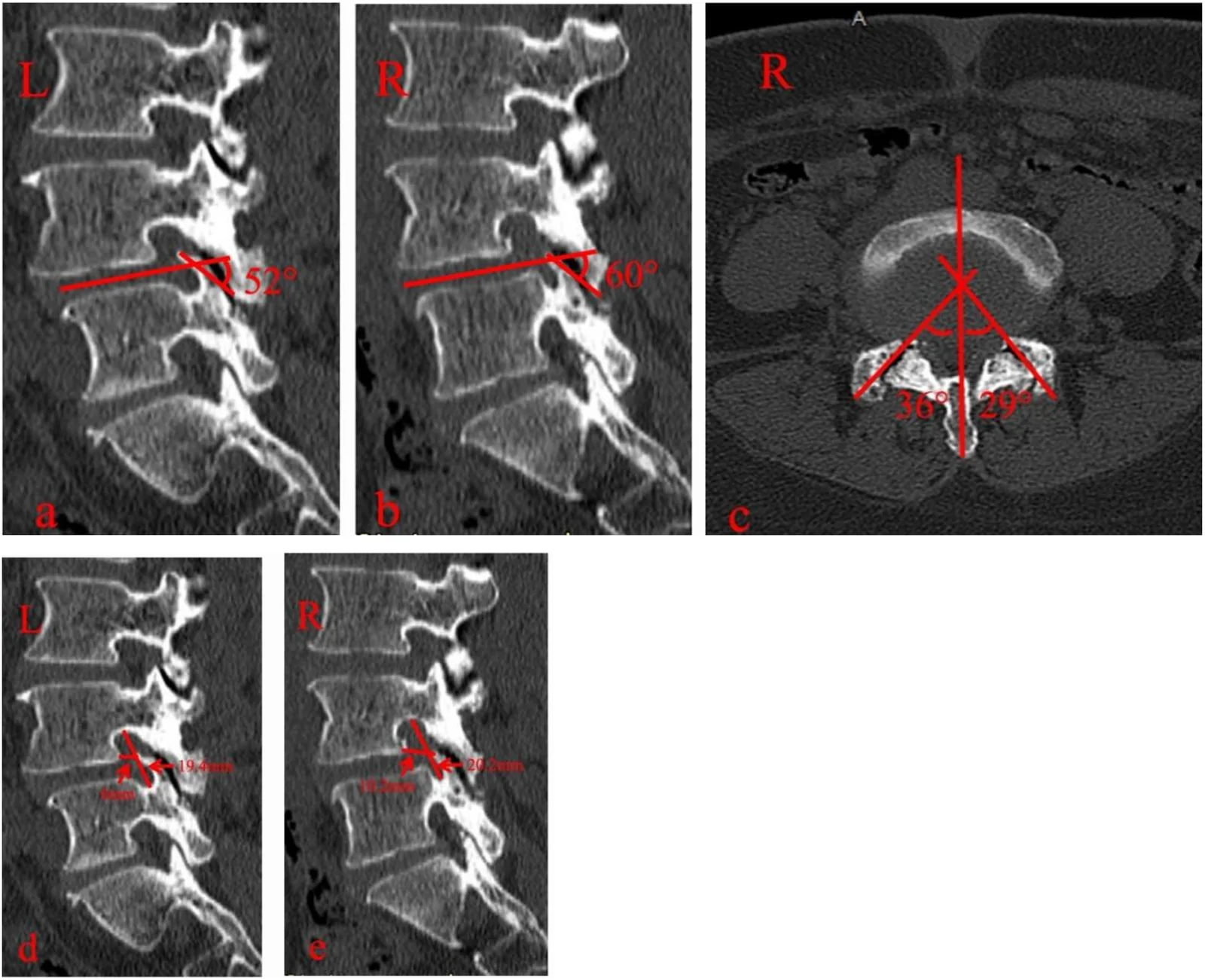
Preoperative CT scans demonstrated L4/5 facet joint degeneration and elongation of bilateral L5 superior articular processes (SAP). The difference of bilateral facet joint angles was 8° in the sagittal plane **(a,b)** and 7° in the axial plane **(c)** The width of the left neuroforamen was smaller than that of the right, while the heights were nearly equivalent **(d,e)**.

Preoperative lumbar radiographs demonstrated grade Ⅰ spondylolisthesis at the level of L4/5 (Figure 1); a preoperative MRI revealed L4/5 disc herniation and right foraminal stenosis secondary to sequestered disc fragment, while the left neuroforamen appeared nearly normal (Figure 2). Preoperative CT scans exhibited L4/5 facet joint degeneration, the elongation of bilateral L5 superior articular processes (SAP), and the secondary reduced width of the neuroforamen on the left side. Facet joint angles were measured according to what was stipulated by Grogan et al. (10) and Ishihama et al. (11) to demonstrate the facet tropism in the axial and sagittal planes, and the height and width of the foramen were measured according to what was prescribed by Liu et al. (12) (Figure 3).

#### Therapeutic intervention

A conventional open TLIF was performed at L4/5 level via a right-sided approach. After the insertion of four fenestrated pedicle screws and bone cement enhancement, the right facet joint and bilateral ligamentum flavum were radically resected, and the spinal canal decompression was accomplished by resecting the medial portion of the left facet joint to visualize the exiting spinal nerve root (L4). Following discectomy, segmental reduction was performed and a 12 × 26 mm cage combined with autologous bone graft was implanted into the disc space for interbody fusion.

#### Development of contralateral symptoms

Preoperative right-sided radicular symptoms were relieved immediately after the primary surgery. However, new-onset anterolateral radiating pain in the left thigh and dysesthesia involving the medial left calf developed on postoperative day 1 (VAS 7). A physical examination showed grade 4 muscle strength of the left quadriceps femoris and an absent left patellar tendon reflex, whereas the right quadriceps strength and patellar tendon reflex remained intact. Perineal sensation and micturition and defecatory functions were intact.

#### Postoperative imaging

**Figure 4:**
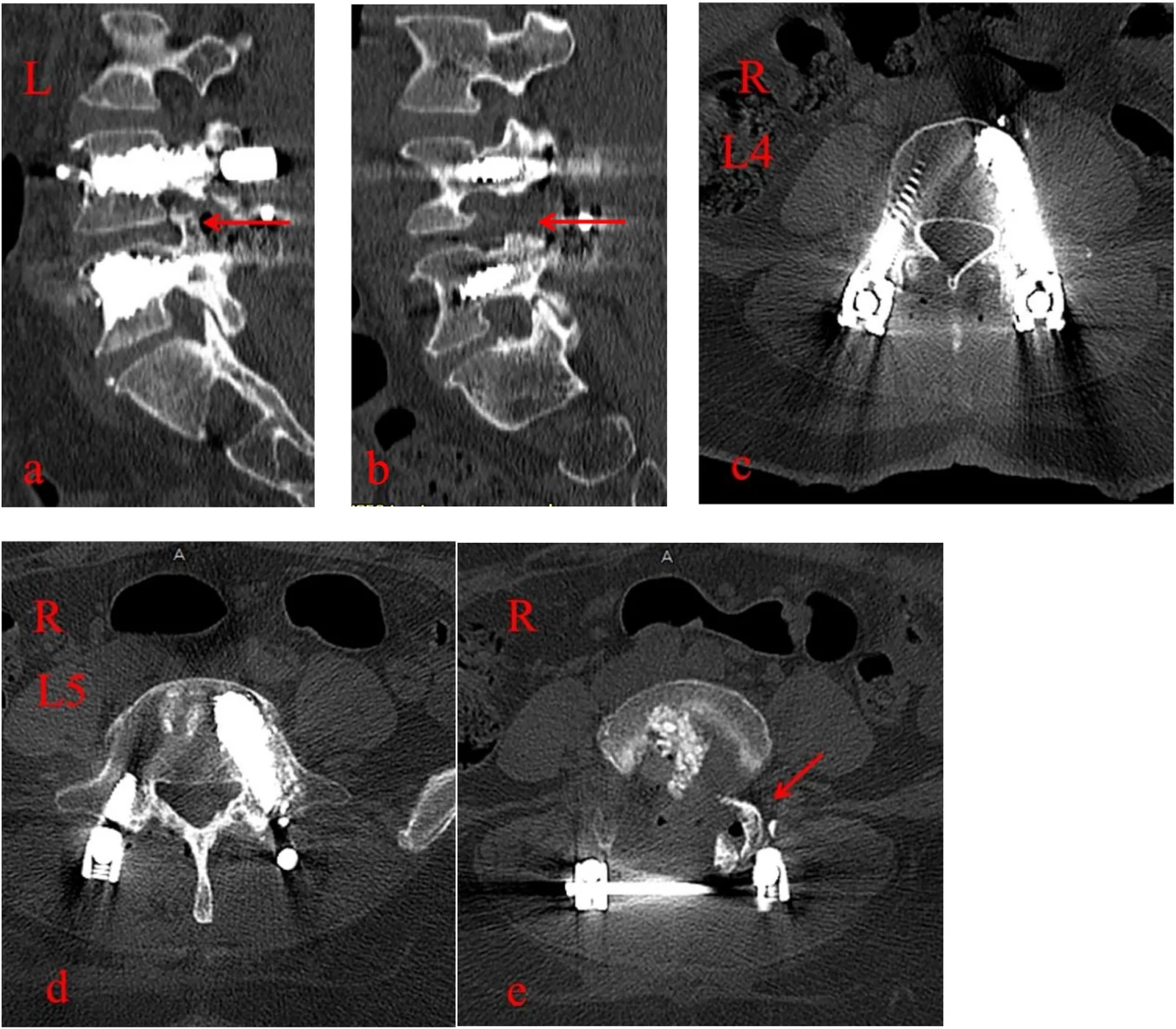
A postoperative CT demonstrated satisfactory segmental reduction and optimal positioning of the interbody cage and pedicle screws with no obvious cement leakage **(a–e)**. Sagittal and axial images showed residual bony tissue of the left SAP resulting in secondary foraminal stenosis **(a,e)**, in contrast to the surgically enlarged neuroforamen on the right **(b)**.

**Figure 5:**
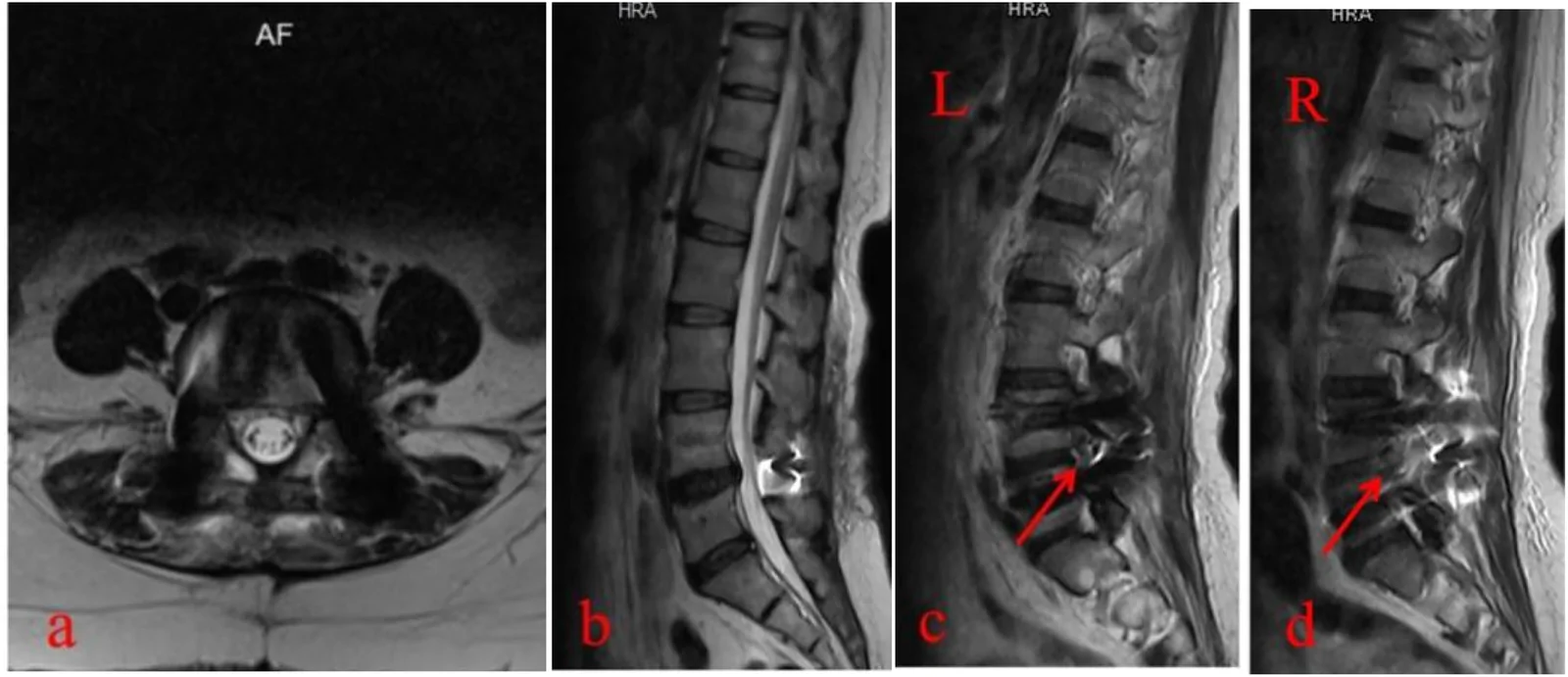
A postoperative MRI demonstrated adequate decompression of the central canal and ruled out epidural hematoma and residual disc–induced neural compression **(a,b)**. The foraminal region showed blurred and irregular signal characteristics, which precluded accurate radiological interpretation **(c,d)**.

A postoperative CT demonstrated satisfactory segmental reduction, optimal positioning of the interbody cage, and pedicle screws with no obvious cement leakage. However, the CT revealed anterior displacement of residual bony tissue of the left SAP, resulting in secondary foraminal stenosis (Figure 4). A postoperative MRI demonstrated adequate decompression of the central canal and ruled out epidural hematoma and residual disc–induced neural compression. The foraminal region showed blurred and irregular image findings, which precluded accurate clinical guidance (Figure 5).

#### Revision surgery

**Figure 6:**
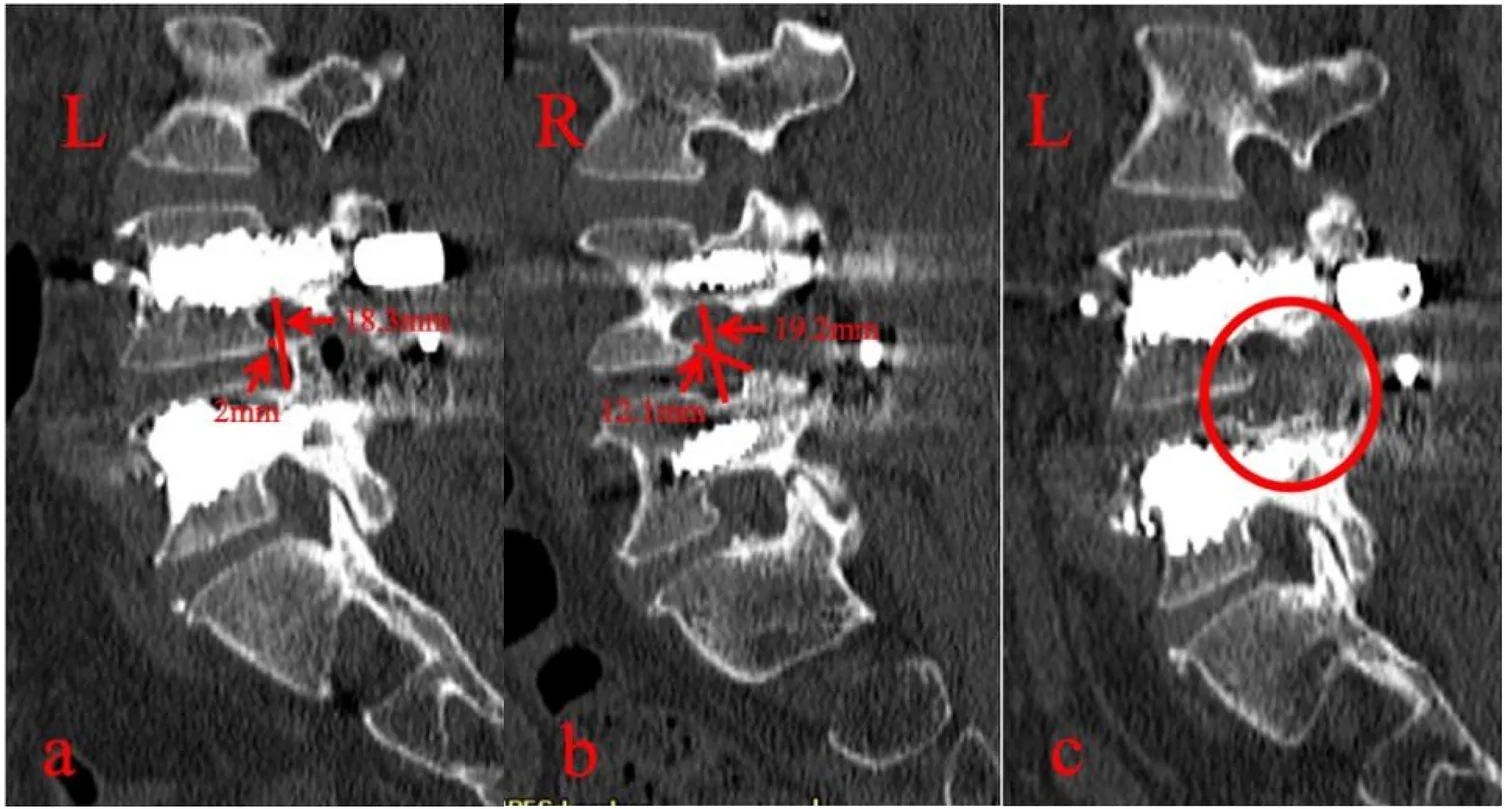
The measurements of the neuroforamina following the primary TLIF revealed reduced width on the left **(a,b)**. The left neuroforamen was surgically enlarged after the revision **(c)**.

The patient responded poorly to conservative treatment such as analgesics, non-steroidal anti-inflammatory drugs, and low-dose steroids. An open revision surgery was conducted 7 days after the primary fusion to decompress the neuroforamen by removing the residual SAP of L5 (Figure 6).

#### Neurological recovery and final follow-up

The left radicular symptoms were relieved following the revision decompression (VAS 3). The patient received routine follow-up at 1, 3, 6, and 12 months after the revision surgery. The strength of the left quadriceps recovered to grade 5 at the 6-month follow-up, and the numbness in the medial aspect of the left calf resolved at approximately 1 year postoperatively (ODI 10). The level of patient satisfaction was favorable. There was no recurrence of radiculopathy and no implant-related complications. A preliminary assessment via dynamic X-rays showed good interbody fusion.

#### Patient's perspective

The patient suffered persistent low back pain and right radiculopathy that impaired daily activities. She was pleased with the immediate resolution of preoperative right-sided symptoms after primary TLIF, yet became anxious after new-onset left leg neural pain developed on postoperative day 1. Conservative management failed to relieve her complaints, and she agreed to revision decompression after a thorough communication. Her left radicular symptoms gradually subsided after the reoperation. At the final follow-up, she reported satisfactory recovery and was content with the overall outcome.

### Case 2

A 56-year-old male free of systemic comorbidities was admitted for a 1-year history of low back pain and radiating pain in the left lower extremity that failed to respond to standard conservative therapy (VAS 8, ODI 76). The pain radiated from the left buttock to the posterolateral thigh and posterior calf. The clinical timeline for the patient is shown in Table 2.

**Table 2:** Clinical timeline for the patient (case 2).

Time point	Key events
Admission day (day 0)	Admitted with a 1-year history of intractable low back pain and left lower-extremity radiating pain refractory to conservative treatment (VAS 8, ODI 76). A physical examination revealed left plantar hypoesthesia and grade 4 left plantar flexor strength, with an absent left Achilles tendon reflex and a positive left straight leg raise test result
Hospital day 1 (X-ray, CT, and MRI performed)	Lumbar radiography demonstrated L4/5 grade Ⅰ degenerative spondylolisthesis (Figure 7); an MRI confirmed L5/S1 disc herniation and mild L4/5 central canal and lateral recess stenosis without obvious foraminal stenosis (Figure 8). A CT revealed elongated L5 superior articular processes (SAPs), reduced right neuroforamen width, and evident facet tropism (Figure 9)
Hospital day 2 (day of primary surgery)	Underwent two-level open TLIF (L4/5 and L5/S1) via a left-sided approach with six pedicle screw fixations
Hospital day 3 (CT and MRI performed Conservative management)	New-onset right anterolateral thigh radiating pain and right medial calf dysesthesia developed (VAS 8). An examination revealed grade 4 right quadriceps and tibialis anterior strength and an absent right patellar tendon reflex. A postoperative CT showed satisfactory spinal alignment and implant positioning, whereas the residual right L5 SAP resulted in secondary right foraminal stenosis (Figure 10). A postoperative MRI confirmed adequate central canal decompression and excluded epidural hematoma and residual disc compression, with indistinct foraminal imaging caused by local hemorrhage and edema (Figure 11). The patient responded poorly to analgesics and non-steroidal anti-inflammatory drugs
Hospital day 5 (day of revision surgery)	Underwent endoscopic L4/5 foraminotomy under local anesthesia for right neuroforaminal decompression (Figure 12). Right radiating pain was markedly alleviated after the revision surgery (VAS 2)
Hospital day 8	The patient was discharged and reported satisfaction with the clinical outcome
Three-month follow-up	Right lower-extremity muscle strength and sensory function fully recovered, with significantly improved functional outcomes (ODI 8)
One-year follow-up	No recurrent radiculopathy or implant-related complications occurred. Dynamic radiographs confirmed solid interbody fusion

#### Physical examination and imaging studies

**Figure 7:**
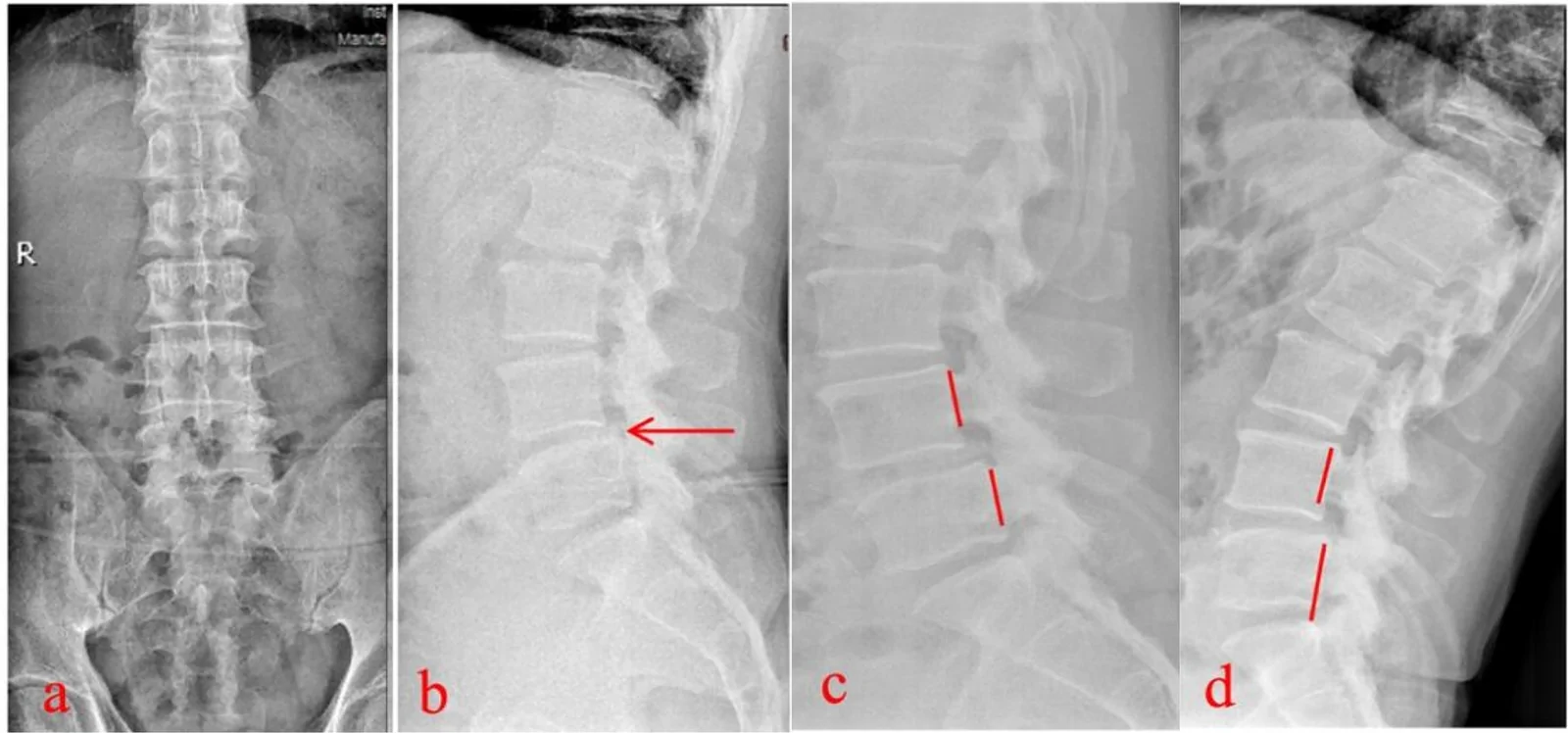
Preoperative lumbar radiographs demonstrated grade Ⅰ spondylolisthesis at the level of L4/5 on the lateral view **(b)** without scoliosis on the AP view **(a)** the lateral dynamic views showed the segmental instability at the L4/5 level **(c,d)**.

**Figure 8:**
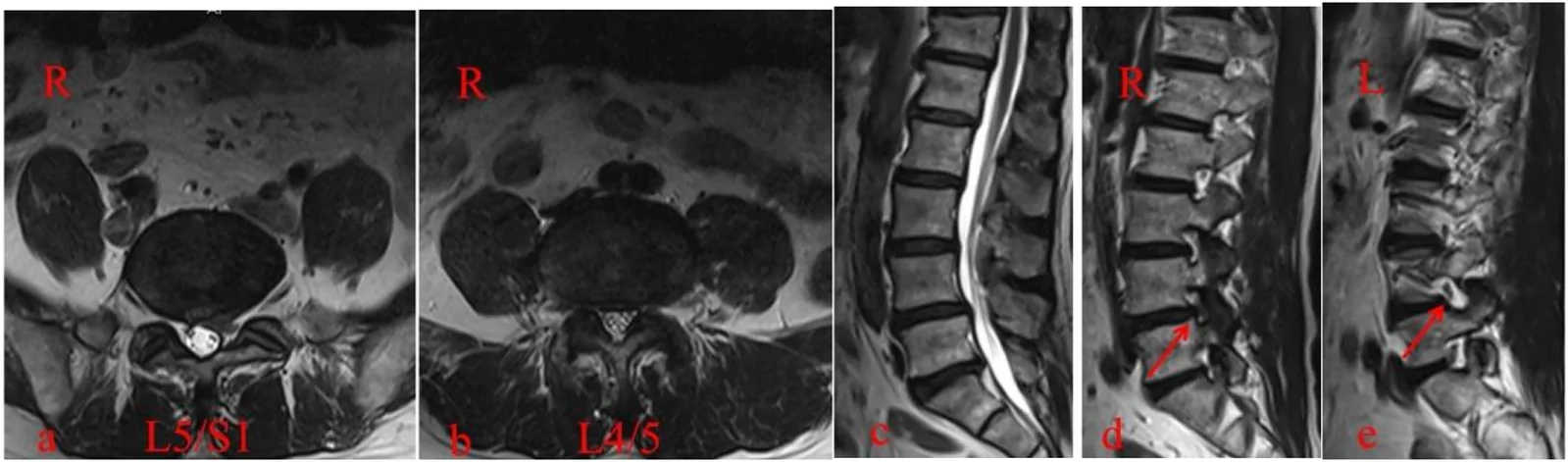
An MRI revealed intervertebral disc herniation at the L5/S1 level and mild central canal and lateral recess stenosis at the L4/5 level **(a–c)**, yet no evident neuroforaminal stenosis was observed on either side **(d,e)**.

**Figure 9:**
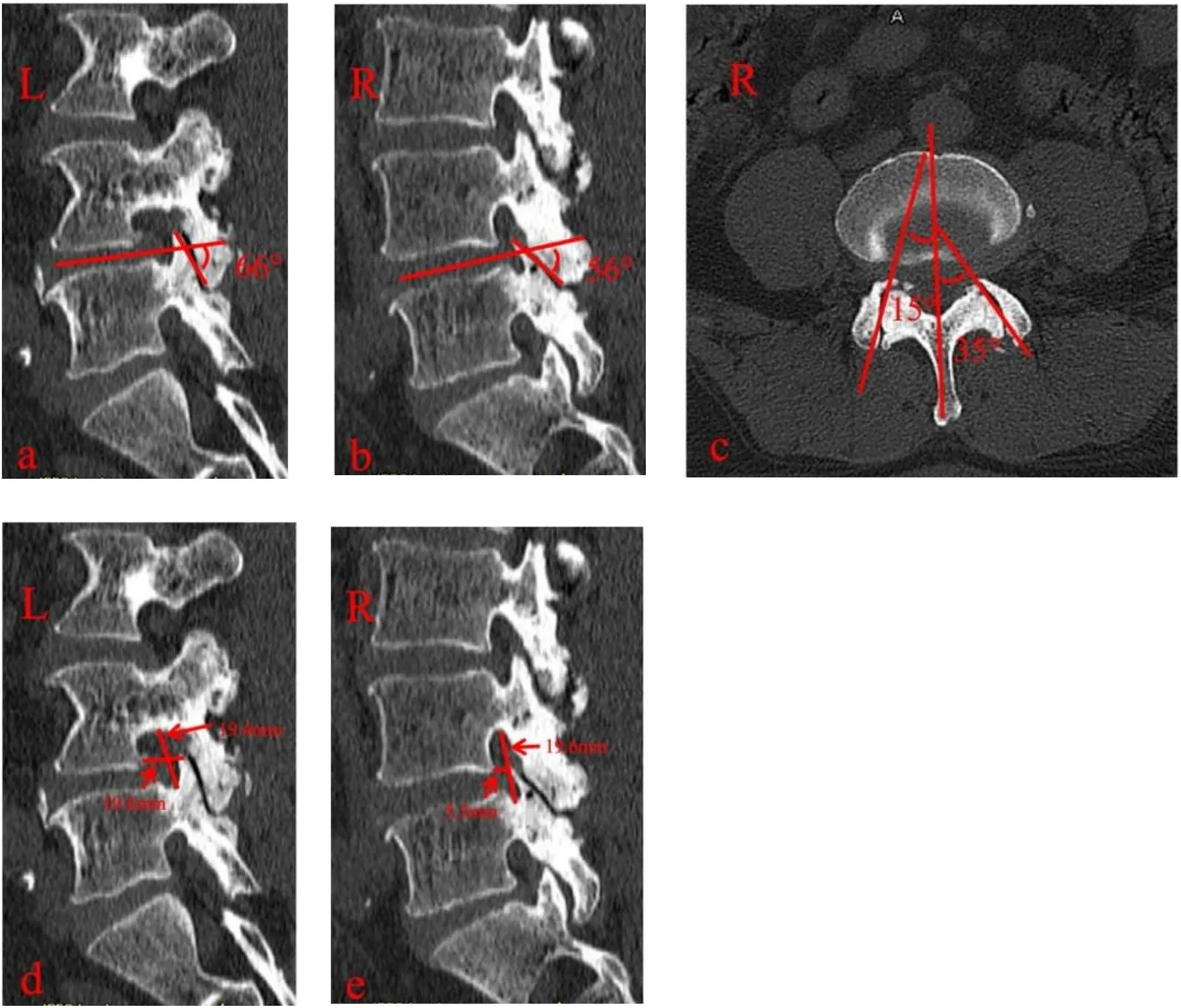
Preoperative CT scans demonstrated L4/5 facet joint degeneration. The difference of the bilateral facet joint angles was 10° in the sagittal plane **(a,b)** and 20° in the axial plane **(c)** The width of the right neuroforamen was smaller than that of the left, while the heights were nearly equivalent **(d,e)**.

A physical examination demonstrated hypoesthesia over the left plantar region and grade 4 muscle strength of the left plantar flexors, whereas the muscle strength of all other bilateral limb muscles was grade 5. The Achilles tendon reflex was absent and the straight leg raise test was positive on the left side. Bowel and bladder functions remained intact. Preoperative radiographs demonstrated grade I L4/5 DS (Figure 7), and an MRI revealed intervertebral disc herniation at the L5/S1 level and mild central canal and lateral recess stenosis at the L4/5 level, yet no evident neuroforaminal stenosis was observed (Figure 8). A preoperative CT showed the elongated L5 SAPs and the secondary reduced width of the neuroforamen on the right side. Facet tropism was demonstrated by measuring the facet joint angles in the axial and sagittal planes (Figure 9).

#### Therapeutic intervention

Two-level open TLIF was performed using six pedicle screws via the symptomatic left-sided corridor. Spinal canal decompression was achieved through complete left facetectomy and medial undercutting of the contralateral right facet joint. Following discectomy and segmental reduction, a 12 × 26 mm interbody cage combined with autologous bone graft was inserted into each disc space at the L4/5 and L5/S1 levels.

#### Development of contralateral symptoms

**Figure 10:**
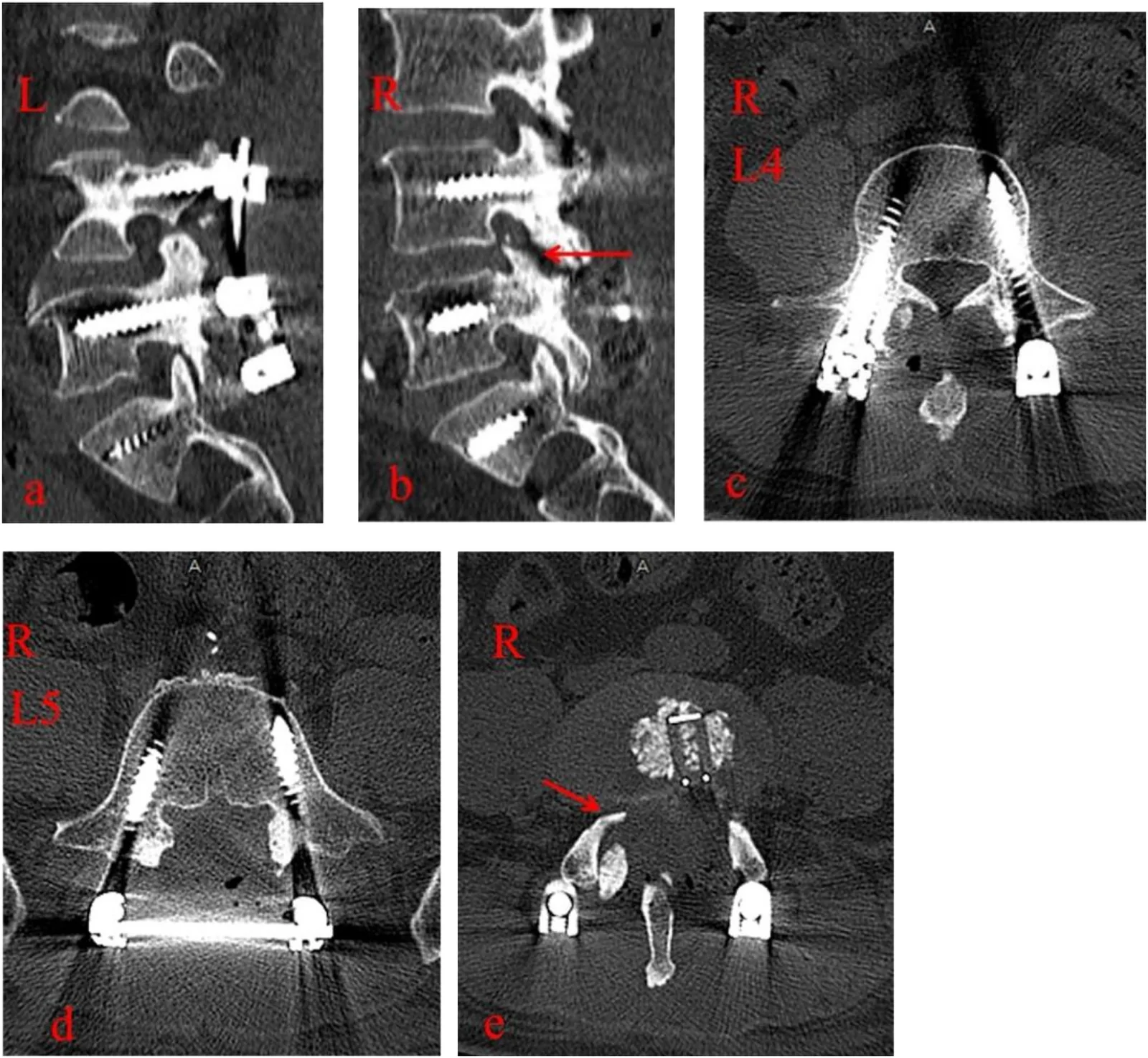
A postoperative CT demonstrated satisfactory lumbar spinal alignment, optimal positioning of the interbody cage, and pedicle screws **(a–e)**. However, the residual right SAP reduced the width of the foramen on the right side **(b,e)**.

**Figure 11:**
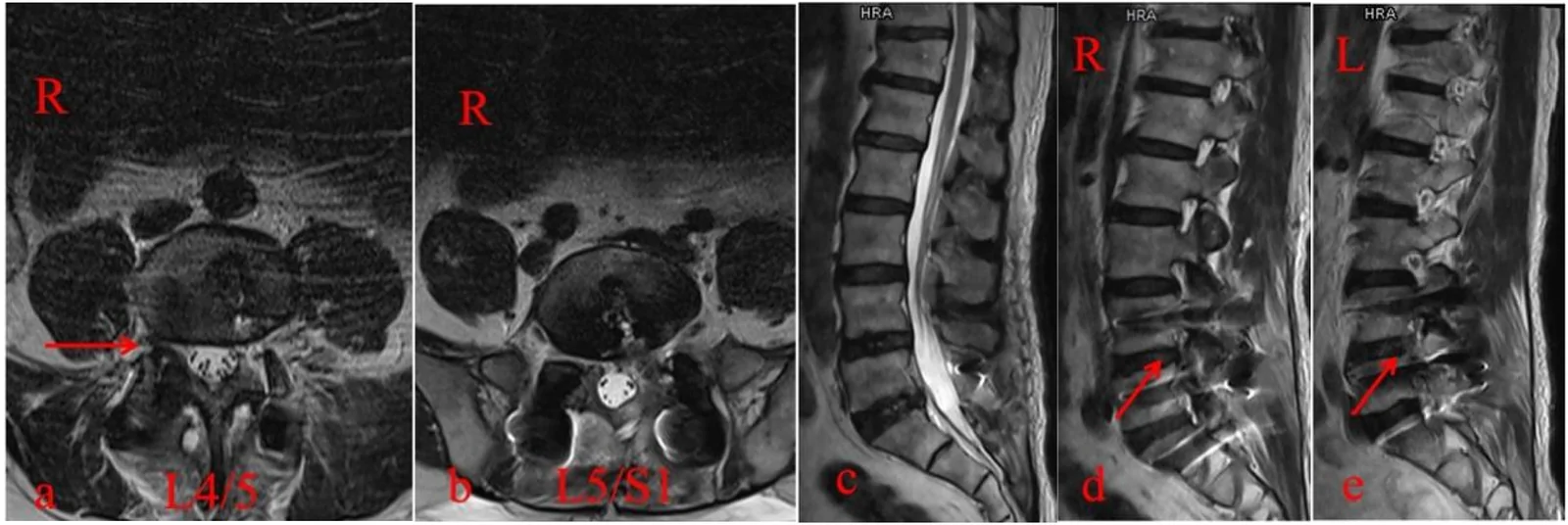
A postoperative MRI demonstrated adequate decompression of the central canal and ruled out epidural hematoma and residual disc–induced neural compression **(a–c)**. The imaging appearance of the neuroforaminal region on MRI was indistinct, making it difficult to identify the cause of nerve root compression **(a,d,e)**.

Postoperatively, the patient's left-sided radicular pain was alleviated, yet new-onset anterolateral radiating pain in the right lower limb and dysesthesia over the medial right calf presented on postoperative day 1 (VAS 8). A physical examination showed grade 4 right quadriceps femoris and tibialis anterior strength (grade 5 on the contralateral side) together with an absent right patellar tendon reflex, whereas the left patellar tendon reflex remained intact. No perineal sensory disturbance or sphincter dysfunction was detected. A postoperative CT demonstrated satisfactory lumbar spinal alignment, optimal positioning of the interbody cage, and pedicle screws. However, the residual right SAP reduced the width of the foramen on the right side (Figure 10). A postoperative MRI demonstrated adequate decompression of the central canal and ruled out epidural hematoma and residual disc–induced neural compression. Owing to focal hemorrhage and edema, the imaging appearance of the neuroforaminal region on MRI was indistinct, making it difficult to identify the cause of nerve root compression (Figure 11).

#### Revision surgery

**Figure 12:**
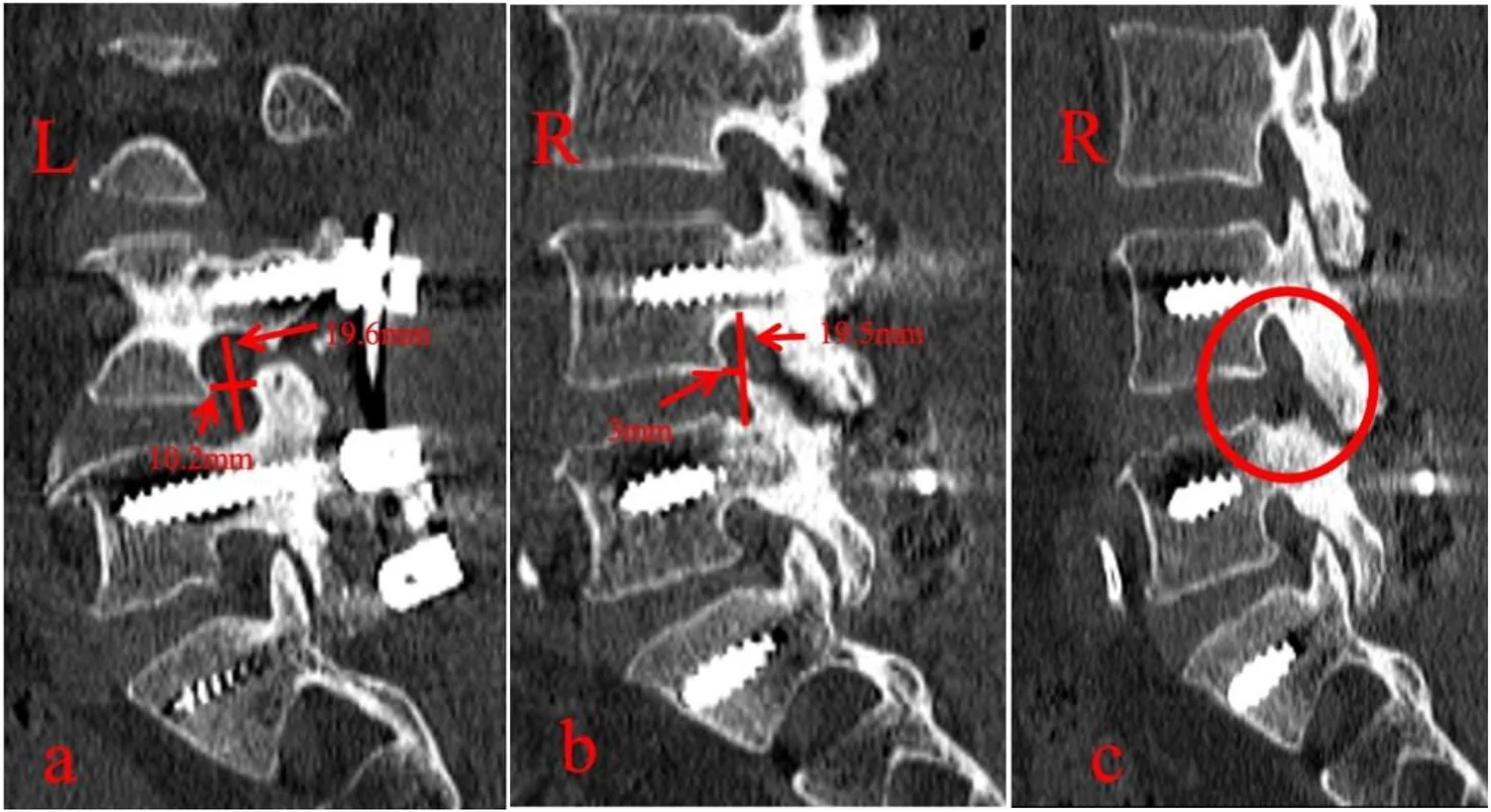
The measurements of the neuroforamina following the primary TLIF revealed reduced width on the right **(a,b)**. The right neuroforamen was significantly enlarged after the endoscopic foraminotomy procedure **(c)**.

Endoscopic L4/5 foraminotomy under local anesthesia was performed after 3 days of unsuccessful conservative management (Figure 12).

#### Neurological recovery and final follow-up

The radiating pain was relieved immediately after the endoscopic decompression (VAS 2). Routine follow-up evaluations were performed at 1, 3, 6, and 12 months postoperatively. The right quadriceps and tibialis anterior muscle strength and lower-extremity sensation returned to normal at the 3-month follow-up (ODI 8). The patient regained daily activity and reported high levels of satisfaction. There was no recurrence of radicular symptoms and no hardware complications. Dynamic radiographs revealed satisfactory interbody fusion at the 1-year follow-up.

#### Patient's perspective

The patient experienced chronic low back pain and left leg radiculopathy limiting daily activities for 1 year. He achieved prompt relief of preoperative left-sided symptoms after primary two-level TLIF but developed unexpected right lower limb neural pain on postoperative day 1, which caused considerable worry. Conservative treatment failed to improve his symptoms, and he consented to endoscopic foraminotomy after adequate consultation. Right radicular complaints resolved rapidly following revision decompression. At the 1-year follow-up, neurological function fully recovered, and he expressed high satisfaction with the overall treatment outcome.

## Discussion

Since Kirkaldy-Willis published his landmark biomechanical research characterizing degenerative spondylolisthesis in 1978 (13), cumulative clinical and biomechanical studies have established that segmental spinal instability and vertebral anterior slippage, induced by progressive intervertebral disc and facet joint degeneration, constitute the core pathogenic drivers of DS (14, 15). TLIF is a standard surgical technique for adult DS, with multiple inherent advantages including expanded neural foramina, improved restoration of segmental lumbar lordosis, enlarged graft contact area, and stable short- and long-term fusion outcomes (16). Reported complications associated with TLIF include intraoperative neurological injury, interbody cage or bone graft migration, dural tear, surgical site infection, heterotopic ossification, postoperative radiculopathy, osteolysis, and vertebral subsidence (17). Contralateral radiculopathy has been recognized by spinal surgeons as a rare adverse event with an overall incidence rate of 2.5%, and multiple heterogeneous pathogenetic explanations have been proposed, such as pedicle screw malposition, newly developed herniated nucleus pulposus, postoperative epidural hematoma (18), decreased postoperative disc and foraminal height (19) or cross-sectional area (20), anterior SAP displacement induced by excessive lordotic reduction (21), preoperative sagittal segmental mobility, and interbody cage positioning (22).

In the two cases described herein, postoperative contralateral radiculopathy was confirmed to result from SAP bony encroachment into the neural foramen after instrumented reduction of spondylolisthesis, leading to mechanical compression of the exiting spinal nerve root. Notably, a preoperative CT imaging identified significant facet tropism in both patients, which possibly contributed to secondary foraminal stenosis. Tropism refers to involuntary orientation by an organism or one of its parts that involves turning or curving by movement or by differential growth, and is a positive or negative response to a source of stimulation. Originating from the biological root of the word tropism, facet joint tropism is conventionally defined as angular asymmetry of bilateral facet joints for quantitative measurement in radiological studies, with a difference of facet joint angle ≥7°in axial plane10 or >5° in sagittal plane11 to facilitate clinical interpretation. Facet articular surfaces deviate in the coronal plane, as facet tropism is generally induced by chronic spinal rotational biomechanical stimuli (23). However, Boden et al. (1) reported that bilateral lumbar facet joints tend to display sagittalized orientation in patients diagnosed with degenerative spondylolisthesis. A sagittal CT reconstruction of our two patients also revealed bilateral sagittal morphological changes of the facet joints compared with adjacent intact spinal segments. The case 2 patient exhibited severe facet angular asymmetry in the axial plane (20°) and sagittal plane (10°), while the asymmetry in the case 1 patient was moderate in the axial plane (7°) and sagittal plane (8°). The facet tropism in the two cases is characterized by a more coronally oriented SAP on the surgical side and a sagittalized SAP on the contralateral side.

Although the correlation between facet tropism and degenerative spondylolisthesis has been widely documented, the causal relationship between these two lesions remains ambiguous. Binder and Nampiaparampil (24) hypothesized that lumbar segments with small facet joints carry elevated risks of developing degenerative spondylolisthesis. Ishihara et al. (25) found that facet joint orientation at the slip level as well as adjacent superior and inferior segments differed significantly between patients with DS and healthy control subjects. Su et al. (26) retrospectively analyzed CT imaging data from 70 patients with DS and 50 patients without lumbar degenerative disorders and concluded that DS is typically accompanied by a severe SAP and articular capsule hyperplasia; these bony and soft tissue lesions may precipitate postoperative nerve root compression after complete intraoperative slip reduction and require targeted attention during surgical decompression. Preoperative CT scans of our two patients exhibited that SAPs of the caudal vertebra exhibited anterior and superior elongation and attenuation to adapt to chronic vertebral slippage, with more prominent morphological changes on the contralateral side of surgical access. Accordingly, we hypothesize that facet tropism and subsequent horizontal foraminal stenosis may serve as the pathological basis for this specific postoperative complication. Cyron and Hutton (27) proposed that relative spinal axial rotation shifts toward the side of the more coronal facet joint, generating excessive mechanical stress on the intervertebral disc annulus fibrosus and facet capsular ligaments. This biomechanical inference aligns with our clinical observations: the primary radicular symptoms developed on the side with a more coronally oriented SAP in both patients, whereas the contralateral sagittalization of the SAP contributes to the foraminal stenosis and revision surgery. Nevertheless, controlled large-cohort clinical studies are required to validate that a coronal SAP predisposes to medial nerve root irritation and that a sagittalized SAP morphology exacerbates foraminal stenosis after a sagittal plane reduction of DS.

TLIF is conventionally performed from the more symptomatic side via unilateral laminotomy and partial facetectomy, while bilateral laminectomy is reserved for patients with severe preoperative bilateral neural compression identified on imaging (28). Although medial undercutting of the contralateral facet joint was routinely implemented in our two cases, the decompression range failed to reach the lateral margin of the neural foramen, representing one major contributor to postoperative contralateral exiting nerve root compression. Given the above findings, adequate contralateral foraminal decompression may be considered intraoperatively even in patients without preoperative contralateral radicular symptoms, particularly among cases complicated with preoperative facet tropism. For open TLIF, foraminectomy or targeted foraminotomy can be readily conducted to fully release the exiting spinal nerve root. During MI-TLIF, tubular retractors can be medially angled to achieve extensive decompression of the spinal canal and contralateral neural foramen (29). For Endo-TLIF, unilateral laminotomy bilateral decompression can be applied for patients with bilateral spinal stenosis (30). However, extensive lateral decompression of the contralateral foramen remains technically challenging during minimally invasive surgeries. Under such circumstances, a systematic preoperative CT review is critical to predict the risk of postoperative contralateral radiculopathy.

Contralateral radicular symptoms are more prone to develop when surgeons pursue complete slip reduction to restore physiological lumbar lordosis and global spinal sagittal alignment, as aggressive reduction exacerbates vertical and horizontal foraminal stenosis in patients with facet tropism. This may explain why DS of Grade II or higher has been linked to contralateral radiculopathy. Such a complication is rare and etiologically perplexing in patients with Grade I DS. The facet tropism may be a predisposing factor for severe reduction-related symptomatic stenosis. Even a slight reduction of Grade I DS in the setting of facet joint pathology (facet tropism) can trigger clinical symptoms. Theoretically, *in situ* fusion could mitigate this specific complication. However, insufficient lumbar lordosis is closely associated with poor long-term postoperative patient satisfaction. Multiple clinical and biomechanical studies have verified that restoration of segmental lumbar lordosis reduces the incidence of adjacent segment degenerative disease (31). Given the well-established clinical value of sagittal alignment reconstruction, spinal surgeons increasingly adopt various techniques to achieve complete reduction of spondylolisthesis. When full reduction is planned preoperatively, surgeons must comprehensively evaluate facet joint morphological alterations and the required range of contralateral decompression.

This unique postoperative complication mainly manifests as radiating limb pain and motor weakness induced by a bony compression of exiting nerve roots, presenting as anterior thigh pain and quadriceps (and possibly tibialis anterior in certain patients) weakness at the L4/5 segment. Because nerve root compression originates from bony osteophytes in these cases, conservative therapy yielded limited efficacy. We therefore recommend timely surgical revision for patients with severe refractory radicular pain and obvious limb motor weakness. Both open foraminectomy and endoscopic foraminotomy could achieve effective decompression, and endoscopic revision is associated with less trauma, smaller surgical wounds, and reduced risk of surgical site infection.

## Conclusion

We report two cases of postoperative contralateral radiculopathy following TLIF for lumbar degenerative spondylolisthesis, with facet tropism possibly representing a predisposing anatomical risk factor. This complication is likely attributable to mechanical compression of the exiting spinal nerve roots exerted by sagittalized SAPs upon full intraoperative slip reduction. A systematic and detailed evaluation of preoperative images is essential to prevent this adverse event from occurring. Once this complication is clinically confirmed, timely consideration of revision surgery is warranted. Endoscopic foraminotomy may be a minimally invasive option in selected patients and in experienced hands.

It should also be noted that this study is based on only two cases and lacks a control group. Therefore, it is hard to establish a causal relationship between facet tropism and postoperative contralateral radiculopathy. Further studies with larger clinical case series are required to draw more definitive conclusions.

## Data Availability

The original contributions presented in the study are included in the article/Supplementary Material, and further inquiries can be directed to the corresponding author.
